# Diagnostic Utility of Canine C-Reactive Protein, Haptoglobin, and 25-Hydroxyvitamin-D in Dogs with Nasal Cavity Disease

**DOI:** 10.3390/ani14192908

**Published:** 2024-10-09

**Authors:** Sarah Rösch, Julia Woitas, Gerhard Ulrich Oechtering

**Affiliations:** Ear, Nose and Throat Unit, Small Animal Department, Faculty of Veterinary Medicine, Leipzig University, 04103 Leipzig, Germany

**Keywords:** nasal discharge dog, nasal tumor, aspergillosis, idiopathic rhinitis, rhinoscopy

## Abstract

**Simple Summary:**

The diagnosis of nasal cavity disease (ND) in dogs typically requires comprehensive diagnostics under general anesthesia including cross-sectional imaging and rhinoscopy. Therefore, dogs with nasal discharge are often only presented for diagnostics after weeks of symptomatic or even antibiotic therapy, even though primary bacterial rhinitis is uncommon. In a previous study, the platelet-to-lymphocyte ratio (PLR) differed significantly between dogs with malignant tumors and benign pathology. To further investigate the value of blood tests for diagnosis, the aim of this second part of the study was to evaluate serum markers for their diagnostic utility and whether they may be helpful in making the diagnosis of ND quickly and cost-effectively. Dogs with additional diseases detected by a whole-body CT scan and blood tests were excluded. No significant differences were found between groups for the here evaluated markers C-reactive protein, haptoglobin, and 25-hydroxyvitamin-D.

**Abstract:**

In this prospective blinded study, canine C-reactive protein (c-CRP), haptoglobin (HPT), and 25-hydroxyvitamin-D (25(OH)D) were investigated for their diagnostic value in 55 dogs with nasal cavity disease (ND). After comprehensive diagnostics including a culture-dependent microbiological examination (ME) of nasal swabs, 17 dogs were excluded due to additionally detected systemic diseases or steroid pre-treatment. Included were 25 dogs with malignant ND (13 carcinomas and 12 sarcomas) and 30 dogs with benign ND (7 benign tumors, 13 idiopathic rhinitis (IR), and 10 others), as well as 10 controls. In none of the 72 dogs with ND was primary bacterial rhinitis diagnosed. Although within the reference interval, compared to the controls, c-CRP was significantly higher in dogs with ND in general and in every subgroup except for benign tumors. Serum HPT concentrations were not different among groups. Compared to the controls, 25(OH)D concentrations were significantly lower (*p* = 0.041) in malignant ND and sarcomas (*p* = 0.025). Despite pre-treatment with antibiotics (40/54; 74.1%), in 23/51 (45%) dogs, the ME was positive. Cultivated bacteria did not differ significantly between nasal diseases. The serum markers were not significantly different regarding the positivity of ME. In conclusion, the investigated serum markers were not clinically useful for the reliable detection of canine ND, as was the ME. Because of the low number of dogs with IR and positive or negative ME, further studies regarding c-CRP are needed in a larger group of IR dogs without concomitant diseases to reliably evaluate its utility in IR dogs with suspected secondary bacterial nasal infection.

## 1. Introduction

The diagnosis of nasal cavity disease (ND) can only reliably be obtained under anesthesia, ideally through a computed tomography (CT) of the head, endoscopy of the upper airways, histopathological examination of tumor biopsies, or nasal mucosa and mycological examination of mucosa swabs [1,2]. For diagnosing ND, the culture-dependent microbiological examination (ME) of a nasal swab was not helpful, as the frequency of positive culture and the type of bacteria are not significantly different among the various nasal diseases [2]. Additionally, primary bacterial rhinitis in dogs is uncommon [1] and not observed in recent studies using more comprehensive diagnostics [2]. It should be noted that the nasal mucosa is physiologically colonized by bacteria [3]. Even in nasal swabs of healthy dogs, 27 bacterial species were identified as normal flora by an anaerobic and aerobic cultivation of nasal cavity swabs [3]. Detected bacteria in dogs with nasal discharge are believed to be secondary to another ND or commensal [4,5,6,7]. However, it has been suggested that bacteria may contribute to the worsening of primary diseases [5], which is of therapeutic interest in dogs with idiopathic rhinitis (IR) of unknown etiology. The results of culture-based ME do not reliably display the bacterial profile that is detected by culture-independent sequencing methods [5]. However, an early diagnosis and appropriate therapy of canine ND are urgently needed because first-line treatment with antibiotics is not indicated and may lead to a time delay or worsening of diseases (e.g., nasal tumors, mycotic diseases, foreign bodies) [8].

Diagnostic examinations under anesthesia are costly, and owners might be afraid of the anesthetic risk, which is why there is a need for easy and inexpensive blood markers for diagnosing nasal cavity disease as well as for monitoring therapeutic success. Therefore, as the first part of a prospective trial, we described in a previous publication the blood values and ratios (e.g., neutrophil-to-lymphocyte ratio [NLR], albumin-to-globulin ratio [AGR], and the platelet-to-lymphocyte ratio [PLR]) [9]. In this previous study, only the PLR was significantly higher in dogs with malignant ND than in those with benign ND or controls [9]. These observations brought forward the question whether other additional serum inflammatory markers in dogs are related to ND and, as such, whether they may be utilized for diagnosis.

During inflammation and tumorigenesis, the concentrations of acute phase proteins (APPs), such as canine C-reactive protein (c-CRP) and haptoglobin (HPT), can increase [10], while other serum biomarkers, such as 25(OH)D, may decrease [11,12]. In malignant tumors, increases in APPs may be caused by major inflammatory reactions due to infectious or non-infectious mild-to-severe inflammation, which has not been observed in benign neoplasia [13]. Even if the acute phase response is generally short-lived, lasting just a few days, it supports the body’s innate immune defenses. In chronic inflammation, elevated concentrations of APPs have also been reported [10]. A study of 48 dogs with ND revealed a significant difference between healthy dogs and dogs with ND in c-CRP as well as in HPT [14]. Furthermore, significantly different levels were detected in HPT between dogs with rhinitis and aspergillosis [14]. In humans, lower serum 25(OH)D concentrations were associated with a higher risk of respiratory infections [15] and allergic rhinitis in children [16]. The recent literature showed that 25(OH)D was significantly lower in dogs with sinonasal aspergillosis [17]. 

After the diagnosis of IR, which is a diagnosis of exclusion after comprehensive diagnostics, treatment with doxycycline or macrolides may ameliorate clinical signs [2,18], although it is reported that these dogs in general respond poorly or only temporarily to antimicrobial treatment [2,18,19,20]. It is not clear whether the antimicrobial treatment of some kind of bacterial infection versus the immunomodulatory or anti-inflammatory effect (e.g., of doxycycline) is leading to a clinical improvement [2,7]. In some dogs, a good treatment response to corticosteroids or cyclosporine has been described [19]. There are no general recommendations for the use of antibiotics in dogs with IR, which is especially of interest in the case of a positive-culture-based ME with a future therapeutic plan of anti-inflammatory agents (e.g., steroids). As c-CRP is used as a marker for a need for antibiotic treatment in humans with rhinosinusitis and suspected secondary bacterial infection [21], we were interested in the utility of this marker in dogs with IR. 

The aim of the present study was to evaluate the diagnostic utility of the serum biomarkers c-CRP, HPT, and 25(OH)D in dogs with ND. Our hypothesis was that these biomarkers would differ between dogs with malignant and benign ND, as well as between subgroups. Furthermore, the concentrations of these inflammatory markers were evaluated with regard to the result of the culture-based ME of a nasal cavity swab.

## 2. Materials and Methods

### 2.1. Selection Criteria and Ethics Approval

The analysis in this present study was performed as part of a prospective, blinded study. As described above [9], enrolled dogs were presented with nasal discharge to the Ear, Nose and Throat Unit of the Small Animal Department at the Faculty of Veterinary Medicine Leipzig University. The protocol for this prospective study was independently reviewed and approved by the ethics committee at Saxony (animal experiment subject to approval TV 02/18, Saxony, Germany, 08/18-07/20). Written informed consent was obtained from all owners.

### 2.2. Examination and Diagnosis–Study Design

Diagnostics of the dogs was performed in a standardized manner, as previously described [9]. The dogs were fasted for 12 h. Prior to anesthesia, additionally to blood samples for general blood cell count and biochemistry, blood for the measurement of serum inflammation markers was collected. A whole-body CT scan with contrast was performed under general anesthesia, as previously described [9]. If nasal tumors were detected, dogs were grouped in regard to tumor size into T-categories 1 to 4 according to the modified scheme of Adams et al. [22]. T-categories were defined as follows: T1 with tumor tissue confined to a nasal cavity; T2 with bone lysis without orbital, subcutaneous, or submucosal mass; T3 with involvement of the orbit, nasopharynx, or subcutaneous or submucosal mass; and T4 with lysis of the cribriform plate [22]. Due to the low number of dogs in the different categories, for evaluation of the here analyzed markers, T1 to T3 were grouped together and compared versus dogs in T4.

To avoid influence of other diseases or drugs on the concentrations of the evaluated markers, dogs with ND additionally suffering from systemic disease or tumors other than those of the nasal cavity, as suspected or observed through whole-body CT or general blood examination, were excluded [9]. Dogs who had received corticosteroids within 14 days prior to presentation were also excluded. To diagnose ND, the bony border of the nasal cavity, nasal conchae, nasal septum, paranasal sinuses, the roots of teeth, the nasopharynx, and middle ears were examined for any pathologies [9]. CT examination was followed by thorough anterograde and retrograde endoscopy of the upper airways without irrigation (HOPKINS^®^ optics, 0°, 2.7 mm, 18 cm and 0°, 4 mm, 30 cm; HOPKINS^®^ optics 120°, 4 mm, 18 cm; Karl Storz, Tuttlingen, Germany) [9]. Continuous video documentation was performed (Image1 S 3-chip FHD camera head TH100, AIDA version 1.3, Karl Storz, Tuttlingen, Germany). Before intranasal manipulation, a maxillary nerve block was placed through an intraoral approach [9]. 

As described before [9], culture-dependent microbiological and mycological examination of nasal mucosa swabs (sterile, from deep within the nasal passages; IDEXX laboratories, Kornwestheim, Germany) were performed. In this microbiological examination by a specialist department in the external laboratory (blinded with regard to the sample origin), the detection of a bacterium in an increased bacterial count was assessed as a “positive” ME by means of a semi-quantitative evaluation [2,9]. ME was not performed in 3 dogs with malignant nasal tumors due to financial constraints of the owners, and in one dog with a malignant nasal tumor, ME was obtained from the regular veterinarian prior to presentation for further diagnostics and had previously been reported as negative. However, this result was not included in the analysis because it was not sampled at the same time of marker concentration detection in the serum samples. 

A histopathological examination of endoscopically taken biopsies of nasal mucosa or of tissue endoscopically classified as tumor tissue was conducted (Antech Lab Germany GmbH, Tierpathologie München, Munich, Germany, with Dr. W. von Bomhard, Dipl. ECVP) [9]. In the statistical evaluation of the histopathological examination of the nasal mucosa in dogs with any kind of rhinitis (idiopathic rhinitis and dogs of the others group), we used—as in the clinical settings—the official diagnosis made in the external laboratory and generally communicated to the examining veterinarian. This histopathological grouping was based on the dominant cell type in the inflammation. The groups were lymphoplasmacytic, necrotizing, neutrophilic, plasmacytic, mycotic, and chronic rhinitis [2]. The latter was defined as inflammatory lesions in combination with remodeling processes (fibrosis, bone remodeling, hyperplasia of the glands, or follicle formation) [2].

### 2.3. Animal Grouping

Based on the test results, dogs were divided into different groups and subgroups, as previously described [9]. The subgroups included dogs with carcinomas (Cs), sarcomas (Ss), benign tumors (BTs, controlled and re-biopsied after two months, repeated histopathologic examination), idiopathic rhinitis (IR, diagnosis of exclusion, separate group to possibly identify markers for therapeutic outcome), and other benign inflammatory diseases (others group, O, including dogs with oronasal defects, nasal foreign bodies, or fungal diseases). For combined group comparison, all dogs with ND were grouped together (total ND), including those with malignant nasal tumors (malignant ND) or benign disease (benign ND, including benign nasal tumor, IR, and others). The duration of follow-up examinations through check-ups under anesthesia and/or telephone calls of dogs with ND included in this study lasted up to two years after the end of the study. However, some dogs were lost during follow-up [9].

### 2.4. Control Dogs

Ten clinically healthy, normocephalic dogs without clinical signs of systemic or nasal cavity disease or any other obvious disease upon blood or whole-body CT examination served as control dogs. The CT of the head was extended to a whole-body CT to assess concomitant diseases. To assess the health of the nasal cavity, nasal mucosa swabs for microbiological and mycological examination were taken. After rhinoscopy, bilateral nasal mucosal biopsies were taken from the ventral turbinate [9]. The included control dogs all showed negative or unremarkable findings in the microbiological, mycological, and histopathological examination. The tissue samples could therefore additionally be used as controls in another study [23]. A physical control examination of the control dogs after 4 weeks revealed no abnormalities. 

### 2.5. Laboratory Tests

As previously described [9], blood samples were collected before anesthesia to prevent the influence of anesthesia on blood markers. The dogs were fasted for 12 h during preparation for anesthesia. Complete blood examination, including blood count, blood chemistry, and electrolytes (heparin plasma and serum), was performed in a blinded fashion at Laboklin (Bad Kissingen, Germany) [9]. 

In this external laboratory, marker concentrations were determined using the Gentian canine CRP immunoturbidimetric assay (Gentian Diagnostics ASA, Moss, Norway) for serum c-CRP [24,25,26,27] and the Tridelta PHASE Haptoglobin Assay (cat. No. TP-801; Tridelta Development Limited, Maynooth, Ireland) for serum haptoglobin concentrations [28]. Serum 25(OH)D concentrations were examined using a chemiluminescence immunoassay (CLIA; device ADVIA Centaur XPT; Siemens Advia Centaur Vitamin D Total Assay, Siemens Healthineers, Erlangen, Germany) validated in the external laboratory for the use in dogs with the following results in intra- and inter-assay variance testing: intra-assay: 2.72% CV and inter-assay: 2.91% CV in the reference range for dogs. The accuracy of the measurements is confirmed by regular participation in the Reference Institute for Bioanalytics (RfB) interlaboratory comparison.

### 2.6. Reference Intervals of Serum Biomarkers 

The upper reference interval provided for c-CRP by the laboratory was 15 mg/L. Values above 15 mg/L were considered elevated. HPT values are given in g/L. For HPT, values up to 3 g/L were considered physiological, and values above this upper reference limit were considered elevated. Serum 25(OH)D concentrations were given in nmol/L. The reference interval provided by the laboratory was 48–350 nmol/L (120–875 ng/mL).

### 2.7. Statistics

As described before [9], statistical analyses were performed using GraphPad Prism (v 9.5.1., La Jolla, CA, USA). Serum c-CRP, HPT, and 25(OH)D data were tested for normal distribution using the D’Agostino and Pearson normality test and the Shapiro–Wilk normality test. All data were found to be not normally distributed. Comparisons of multiple non-parametric group data were performed using the Kruskal–Wallis test and Dunn’s multiple comparisons test. Results are presented as median values with an interquartile range (IQR). For post-hoc comparisons of two nonparametric groups, the Mann–Whitney test (two-tailed) was used [14]. Statistical significance was set at a *p*-value ≤ 0.05. Spearman non-parametric correlation (Spearman correlation coefficient = rs) was used to evaluate the correlation between serum markers as quantitative variables. Marker concentrations in regard to culture-dependent ME, distinct bacteria, or T-categories were not normally distributed and statistically compared in regard of the size of data sets with the Mann–Whitney test (two-tailed) or Kruskal–Wallis test with Dunn’s multiple comparisons test. To evaluate the effect of prior antibiotic treatment (<4 weeks or more than 4 weeks before presentation) compared to no antibiotic pre-treatment, the chi-square test (two-sided with a 95% confidence interval) was performed.

## 3. Results

In total, 55 of 72 dogs were included, comprising 13 dogs with nasal carcinomas, 12 dogs with nasal sarcomas, 7 dogs with benign nasal tumors, 13 dogs diagnosed with chronic IR, and 10 dogs with various benign inflammatory diseases, which were assigned to the “others” group (for diagnoses, see Supplementary Table S1). In none of the 72 dogs with nasal disease was a primary bacterial rhinitis detected. Therefore, the group with malignant ND included 25/55 dogs (carcinomas and sarcomas; 45%), and the group with benign ND included 30/55 dogs (benign tumors, IR, and the other group; 55%) [9]. The clinical nasal signs such as sneezing and/or nasal discharge in the 55 included dogs of the present study had been observed for a median duration of 5 months (IQR: 2–10). There were no statistically significant differences in the duration of clinical signs between the subgroups (carcinomas: median 6 months [IQR: 3–10.5], sarcomas: median 3.5 months [2–7.5], benign tumors: median 5 months [IQR: 4–7], IR: median 8 months [IQR: 2–18], other: 3 months [IQR: 1.4–7.5]). The inflammatory serum markers c-CRP, HPT, and 25(OH)D were recorded for all subgroups (Table 1).

### 3.1. Correlation between the Serum Inflammatory Markers

A significant negative correlation was detected between c-CRP and 25(OH)D (negative correlation; rs = −0.273; *p* = 0.044). The comparison of the concentrations of the inflammatory markers in the present study with the previously calculated NLR [9] revealed a significant positive correlation of the NLR and c-CRP (rs = 0.404; *p* = 0.003).

### 3.2. Haptoglobin

HPT concentrations (all values in the reference interval up to 3 g/L; Table 1) were not significantly different among dogs with nasal cavity disease (median: 1.6; IQR: 1.1–1.8 g/L) or among the different groups compared with control dogs (median: 1.4; IQR: 0.3–1.7 g/L). 

### 3.3. 25-Hydroxyvitamin-D 

**Figure 1:**
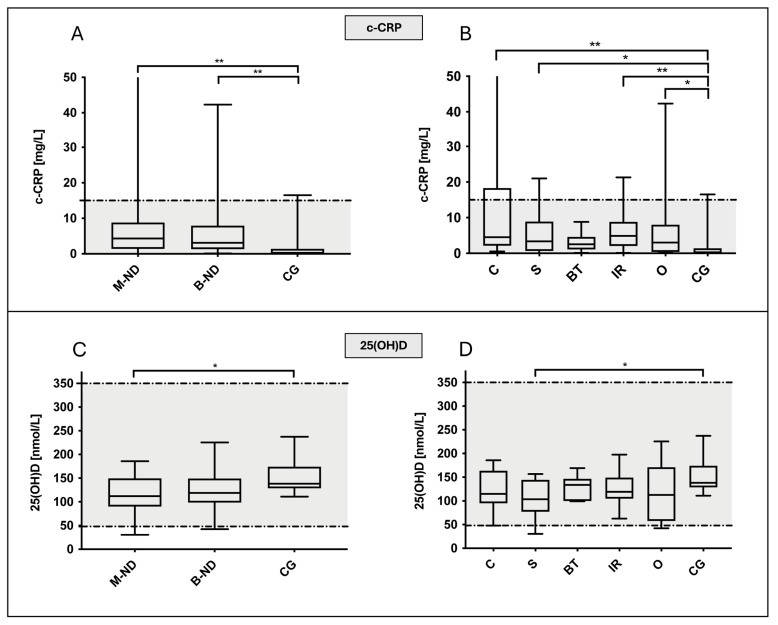
Serum concentrations of c-CRP [mg/L] and 25(OH)D [nmol/L] in 55 dogs with nasal cavity disease and control group (CG). The gray marked area in all four boxplot diagrams shows the reference intervals of the respective parameter shown (c-CRP up to 15 mg/L, 25(OH)D 50–350 nmol/L). (**A**,**B**) Serum c-CRP was significantly increased in dogs with both benign (*p* = 0.006; B–ND) and malignant nasal cavity diseases (*p* = 0.002; M-ND) compared to the CG. This significant difference was due to significantly increased c-CRP levels in dogs with carcinomas (Cs) and idiopathic rhinitis (IR) but also in dogs with sarcomas (Ss) or other nasal diseases (Os), when compared to CG. Canine CRP values of dogs with benign tumors (BTs) were not significantly different from those in the CG. (**C**,**D**) Concentration of 25(OH)D was significantly decreased only in dogs with malignant neoplasia (M-ND) when compared to control animals (*p* = 0.041), with a particularly strong difference observed for dogs with sarcomas (*p* = 0.025). Data are shown in box and whisker plots. Upper and lower boxes represent the 25th and 75th percentiles (lower whiskers = minimum, upper whiskers = maximum values), respectively, and the line represents the median. The asterisks indicate the statistical significance with * = *p* ≤ 0.05, ** = *p* ≤ 0.01.

Median 25(OH)D concentrations of the different groups were within the reference interval of 48–350 nmol/L, except for two single values that were below the reference interval (one dog with nasal sarcoma at 30.3 nmol/L and one dog of the others group with 42.4 nmol/L, Table 1). The 25(OH)D concentrations were significantly lower (*p* = 0.041) in dogs with ND (median: 118, IQR: 94.6–149.3 nmol/L) than in controls (median: 138.4, IQR: 128.7–174.3 nmol/L; Figure 1). While no significant difference was detected between control animals and dogs with benign ND (median: 119, IQR: 98.6–149.3 nmol/L), compared to control dogs, 25(OH)D was significantly decreased in dogs with malignant ND (median: 112, IQR: 90.3–149.5 nmol/L; *p* = 0.041). Dogs with sarcomas, but not dogs with carcinomas, had significantly lower 25(OH)D levels compared to CG (*p* = 0.025).

### 3.4. Canine C-Reactive Protein

The median c-CRP values of the different groups were within the reference interval below 15 mg/L, except for single values per group (Table 1). However, c-CRP was significantly higher in dogs with ND in general (median: 4.1; IQR: 1.4–7.9 mg/L) compared to controls (median: 0.2; IQR: 0–1.4 mg/L; *p* = 0.001; Figure 1). Similarly, c-CRP values of dogs with malignant (median: 4.3; IQR: 1.4–8.8 mg/L; *p* = 0.002) and benign ND (median: 3.1; IQR: 1.3–8 mg/L; *p* = 0.006) were both significantly increased compared to the control group, although they were not significantly different from each other. Further subgroup comparisons showed significantly higher c-CRP concentrations in dogs with nasal carcinomas (*p* = 0.002) and IR (*p* = 0.009) than in the control group but also in dogs with nasal sarcomas (*p* = 0.031) and in those of the others group (*p* = 0.033). Compared to the CG, c-CRP levels in dogs with benign tumors were not significantly increased.

### 3.5. Culture-Dependent Microbiological Examination

**Figure 2:**
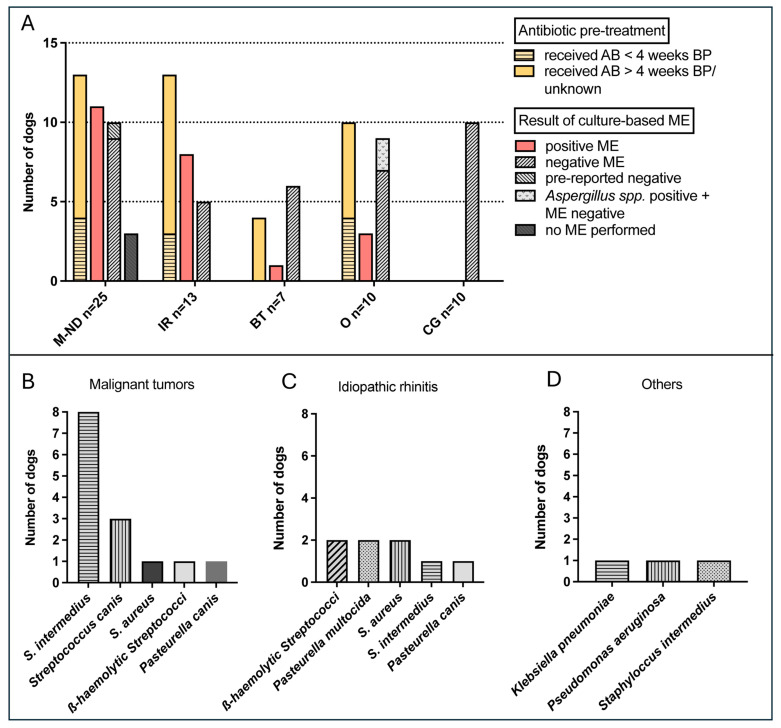
Results of culture-dependent microbiological examination (ME) in 51 dogs with nasal cavity diseases in contrast to the dogs of the control group (CG). (**A**) Despite pre-treatment with antimicrobials (AB), a positive culture-dependent ME was detected in 11/21 dogs (44%) with malignant nasal diseases (M-NDs; sarcomas and carcinomas; n = 25; 3 not performed and 1 only pre-reported negative) and 8/13 dogs (61.5%) with idiopathic rhinitis (IR). Only 1/7 dogs (14.3%) with a benign tumor (BT) had a positive ME, and only 3/10 dogs (30%) in the others group (in 2/3 dogs with *Aspergillus* spp. infections ME was negative). AB = antibiotic; BP = before presentation. In (**A**), the antibiotic pre-treatment is split into treatment less than 4 weeks before presentation (<4 weeks BP) and/or antibiotic treatment carried out before that time (>4 weeks BP). In one dog with carcinoma, no antibiotic history could be discovered. (**B**–**D**) Results of culture-dependent ME show non-significantly different cultivated bacteria. Graphs modified from Rösch et al., 2019 [2].

Antibiotic history was available for 54 of 55 dogs. Antibiotic pre-treatment for nasal disease was reported in 40/54 dogs (74.1%; Figure 2A)—with 13/13 dogs of the IR group, 13/24 dogs with malignant tumor (in one dog, information was not available [n.a.]), 10/10 dogs of the others group, and 4/7 dogs of the group with a benign tumor (Figure 2A). The administered antibiotics were amoxicillin clavulanic acid (n = 10), amoxicillin (n = 4), doxycycline (n = 8), unknown antibiotic (n = 11), enrofloxacin (n = 2), marbofloxacin (n = 5), and cefalexin and clindamycin in one dog each.

A positive culture-dependent ME was reported in 11/21 dogs (44%) with malignant nasal tumors (sarcomas and carcinomas; information was not available [n.a.] in four dogs including one dog with a pre-reported negative ME) and in 8/13 IR dogs (61.5%). In benign tumors, only 1/7 dogs (14.3%) had a positive ME and only 3/10 dogs (30%) in the others group (in 2 of 3 dogs with *Aspergillus* spp., infection ME was negative; Figure 2A). 

The effect of antibiotic pre-treatment on the result of ME was evaluated, after excluding dogs with positive results for *Aspergillus* spp. in combination with a negative ME due to an unknown influence of *Aspergillus* spp. on the ME result, as well as the dogs for which no ME result was available. There was no statistically significant correlation between pre-treatment with antibiotics and the negativity of ME between dogs that had received antibiotics less than 4 weeks prior to presentation (9/54 dogs, 4 positive and 5 negative ME) and dogs that had received antibiotics more than 4 weeks prior to presentation (27/54 dogs, 14 positive and 13 negative ME; chi-square test, *p* = 0.819). Additionally, there was no statistical difference between these pretreated dogs that had received antibiotics in general (n = 36, 18 positive and 18 negative ME) and the dogs without pre-treatment with antibiotics (n = 12, 5 positive and 7 negative ME; chi-square test, *p* = 0.617).

Cultivated bacteria did not differ significantly in various nasal diseases. They are shown in Figure 2B–D, with *S. intermedius* most often cultivated in dogs with malignant nasal tumors. Serum c-CRP values were not statistically different in dogs with distinct detected bacteria such as, e.g., *Pasteurella multocida* or *Staphylococcus aureus*. 

**Figure 3:**
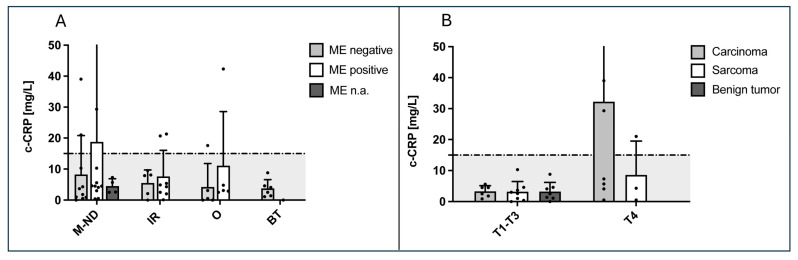
Serum c-CRP depending on positivity of culture-dependent microbiological examination (ME) in nasal cavity disease (ND) and depending on size of nasal tumors (T-categories according to Adams et al. [22]). (**A**) There were no significant differences between c-CRP concentrations in dogs with different nasal cavity diseases and positivity of culture-dependent ME (e.g., IR dogs: median c-CRP in positive ME 4.6 mg/L [IQR: 2.13–16.88] with highest values in dogs with lymphoplasmacytic inflammation/rhinitis and *Pasteurella multocida* and *S. aureus*; median c-CRP in negative ME 7.9 mg/L [IQR: 1.05–8.8]; malignant nasal disease (M-ND): median c-CRP in positive ME 4.5 mg/L [IQR: 3–10.3] with the highest value in *Streptococcus canis*; median c-CRP in negative ME 2.75 mg/L [IQR: 0.8–12.98]). (**B**) Canine CRP values in dogs with carcinomas, sarcomas, and benign tumors (BTs) with size of T1–T3 categories (6 carcinomas, 8 sarcomas, and 7 BT) are displayed versus dogs of T4 category with lysis of the cribriform plate (7 carcinomas and 3 sarcomas). There was no significant difference between c-CRP concentrations in different T-categories in dogs with different malignant tumors. (**A**,**B**) The dashed lines in both graphs indicate the upper reference interval of c-CRP at 15 mg/L.

The positivity of ME revealed no significant differences in the above-mentioned biomarkers (data are exemplarily shown for c-CRP in Figure 3A). Even if median c-CRP concentrations were higher in dogs with positive ME in the groups malignant tumor (*p* = 0.63), idiopathic rhinitis (*p* > 0.99), and others (*p* = 0.25), there was no statistically significant difference.

### 3.6. Excluded Dogs

In total, 17 of 72 dogs were excluded after workup for nasal cavity disease due to, e.g., one or more of the following recognized diseases or due to actual treatment with steroids (n = 5) for one of these underlying chronic diseases. Systemic diseases included polyarthritis or osteoarthritis (n = 5), pulmonary fibrosis, infectious/bacterial pneumonia (n = 2, one dog was diagnosed based on bronchoalveolar lavage results and another based on the clinical signs of fever and pneumonia responsive to antibiotics), mast cell tumor, tumor ventral to the urinary bladder, leishmaniasis (n = 2), structural abnormalities of the liver parenchyma (n = 3), hepatic shunt, chronic pancreatitis, acute gastroenteritis, inflammatory bowel disease, and protein-losing enteropathy. Excluded dogs would have been assigned to the following groups with respect to nasal cavity disease: five dogs in the idiopathic rhinitis group, six dogs in the others group (3/6 dogs with primary SNA), three dogs in the malignant nasal tumor group, and one dog in the benign tumor group. In two dogs, nasal signs were most likely due to rhinitis secondary to the systemic disease of leishmaniasis and, therefore, would not have been categorized into any of the local nasal cavity disease groups.

In general, the c-CRP values of the excluded dogs (see Table 1) were not elevated, with the exception of six dogs (two IR dogs with lung disease, one of the others group with dental disease and lung disease, one dog with malignant nasal tumor in addition to suspected metastases, one dog with benign tumor due to a high-grade oronasal fistula, and one dog with leishmaniasis). Canine CRP concentrations were not significantly increased in this group of dogs in contrast to dogs with nasal cavity diseases but from healthy dogs (Kruskal–Wallis test; *p* = 0.0045; Table 1). Haptoglobin and 25(OH)D concentrations were within the reference interval in all dogs according to our external laboratory and did not differ statistically significantly from dogs with nasal cavity disease or controls (Table 1).

The following information can be emphasized in the context of the subsequent discussion of the results. The three dogs with primary SNA were excluded due to polyarthritis in addition to permanent treatment with prednisolone, due to a lack of whole-body CT or severe osteoarthritis under treatment with mavacoxib. In all three dogs, no increased c-CRP (6.9 mg/L, 8.5 mg/L, and 4.3 mg/L) or haptoglobin (1.45 g/L, 1.14 g/L, and 1.28 g/L) was detected, which is comparable to the results in the dogs of the others group. However, the 25(OH)D concentrations were low (125.8 nmol/L, 53.47 nmol/L, and 52.48 nmol/L).

The five dogs with idiopathic rhinitis were excluded because three of them had lung disease (infectious pneumonia n = 2 and pulmonary fibrosis n = 1), the fourth had inflammatory bowel disease in addition to previous treatment with corticosteroids, and the fifth had pancreatitis in addition to abnormalities of the liver parenchyma. In both dogs with infectious pneumonia, c-CRP was increased at 38.4 mg/L and 25.5 mg/L, respectively, in contrast to the other three IR dogs, in which c-CRP was 0 in the dog with liver abnormalities or in the reference interval (n = 2). Haptoglobin was in the reference interval (1.42 g/L and 1.86 g/L) in the two dogs with pneumonia, as well as in the other IR dogs, as was 25(OH)D.

### 3.7. T-Category of Nasal Tumors

Furthermore, as there was no significant difference between marker concentrations and tumor type, markers were evaluated in regard to tumor size (T-category; T1 to T3 versus T4 with cribriform plate lysis) in malignant tumors. Although there were higher median values in c-CRP in carcinomas and sarcomas in T-category 4 noted, they were not significantly different (carcinomas: *p* = 0.093; sarcomas: *p* = 0.497; Figure 3B). For the reason of completeness, evaluating the influence of tumor size on c-CRP concentrations, Figure 3B shows values of benign tumors, which, however, only had a size up to T-category 3.

### 3.8. Histopathological Examination

**Figure 4:**
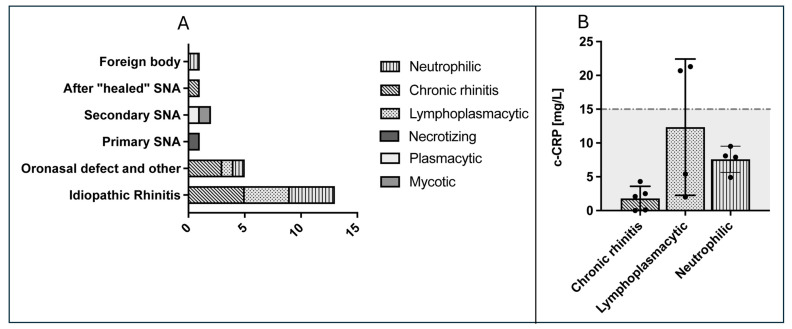
Histopathological examination results in dogs with different kinds of rhinitis. (**A**) Rhinitis with different predominant inflammatory cells was detected. The main cell type communicated by the external laboratory was used for grouping as follows: lymphoplasmacytic, necrotizing, neutrophilic, plasmacytic, mycotic, and chronic rhinitis. The latter was defined as inflammatory lesions in combination with remodeling processes (fibrosis, bone remodeling, hyperplasia of the glands, or follicle formation). SNA = sinonasal aspergillosis. (**B**) Canine CRP (c-CRP) in correlation to different types of inflammation of nasal mucosa in dogs with idiopathic rhinitis is shown. The dashed line indicates the upper reference interval of c-CRP at 15 mg/L. In neutrophilic rhinitis, c-CRP values were not higher than in other types of rhinitis. Graph A modified from Rösch et al., 2019 [2].

Lymphoplasmacytic inflammation was seen in some dogs with IR but also in one dog with oronasal defect (Figure 4A). Neutrophilic inflammation was mostly observed in dogs with IR. In these dogs, the ME was negative and c-CRP within the reference interval (Figure 4). Numerically higher c-CRP values were noted in dogs with lymphoplasmacytic inflammation (not significantly different).

## 4. Discussion

The acute phase proteins c-CRP and HPT and the serum marker 25(OH)D were not significantly different among the groups of dogs with ND in the present study. Therefore, they appear to not be clinically useful for the reliable detection or therapy monitoring of ND in dogs. This result is in line with one previous study in which no high-grade abnormalities in the blood count or blood chemistry in general or between the different groups of dogs with ND were detected [9]. Regarding blood ratios, only the platelet-to-lymphocyte ratio (PLR) was significantly higher in dogs with malignant nasal cavity tumors compared to dogs with benign, inflammatory diseases or to control dogs [9]. 

In the present study, the c-CRP median value of dogs with ND in general of 4.1 mg/L [IQR: 1.4–7.9] was overall in the reference interval with only individual values outside the upper reference limit of 15 mg/L. Therefore, the median c-CRP level of this present study was lower than in another study of c-CRP concentrations in canine ND (aspergillosis: median: 13.4 [IQR: 2–82]; rhinitis: median: 4.8 [IQR: 1–44]; neoplasia: median: 14.8 [IQR: <0.78–69]; healthy dogs: median: 1.5 [IQR: <0.78–9.6]) [14]. The reason for the difference in c-CRP concentrations in both studies could be the measuring method [29]. In the cited study, an immunoturbidimetric assay for canine CRP on a MIRA (Roche Diagnostics) was used [14,30,31] compared to the Gentian canine CRP immunoturbidimetric assay used in this present study [24,25,26,27]. Another reason for the different c-CRP concentrations in dogs with nasal cavity diseases in the two studies could be additional or underlying undetected diseases in the dogs included in the cited study [14]. However, based on the data of the present study, we can conclude that c-CRP levels are not extremely increased in dogs with ND. One reason could be that dogs with nasal cavity diseases are most often not presented in the acute phase but in the late course of the disease (in the present study, the median duration of nasal signs in the 55 included dogs was 5 months [IQR: 2–10]). However, it should be noted that an increase in APPs has also been described under chronic inflammation [10,32]. Another reason could be that ND in dogs does not result in a significant acute phase response, which is what has to be evaluated in further studies evaluating the APPs in dogs with acute clinical nasal signs. Canine CRP, therefore, still can be used for the monitoring of the therapy response of additional or subsequent diseases where c-CRP values are much higher and where c-CRP has been proven as a reliable marker for therapy response. Examples are sepsis [33], SRMA [33], or bronchopneumonia (e.g., with c-CRP: 121 mg/L [IQR 68–178 mg/L]) [34]. This is clinically helpful, as it is known that the aspiration of nasal secretions and secondary bronchopneumonia may occur consequently in dogs with idiopathic rhinosinusitis. Two excluded dogs of the present study with IR (long-term clinical signs of nasal cavity disease) and acute clinical signs of bacterial pneumonia showed increased c-CRP concentrations, supporting this hypothesis.

As reported before, this present study proved that a high number of positive culture-dependent MEs was obtained in the different groups of dogs with nasal cavity disease and that there were no differences between bacterial species [2]. The results of the ME are, therefore, not diagnostic in dogs with ND. According to the literature, the highest number of positive MEs was obtained in the group IR [2]. Additionally, in dogs with malignant nasal tumors, *S. intermedius* was cultivated most frequently [2]. 

Since the use of conventional antibiotics does not lead to long-term therapeutic success in dogs with IR [20], the use of antibiotics in some IR dogs is being discussed. In human medicine, even though in 60% of patients with acute rhinosinusitis bacteria are detected, most cases resolve spontaneously [21]. Additionally, CRP has been shown to be a useful test for the diagnosis of bacterial sinusitis in humans and helped to reduce antibiotic prescriptions in patients with acute rhinosinusitis [21]. This diagnostic benefit could not be verified in the present study for dogs with IR, as the c-CRP value recorded in this study did not statistically significantly differ between dogs with positive and negative ME. A limitation, however, is the small number of dogs with IR and thus with positive ME, which is why a larger number of dogs with IR has to be evaluated after the exclusion of secondary bronchopneumonia or other diseases in order to prove this result. 

The type of mucosa inflammation in IR dogs or the size of malignant and benign tumors in the dogs of the present study were discussed by the authors of the present study as additional influencing factors on the serum concentration of c-CRP. Interestingly, c-CRP concentrations were numerically higher in lymphoplasmacytic rhinitis than in neutrophilic. However, the number of animals is too small to conclusively prove this finding. Regarding tumor size, classification into T-categories can be helpful to establish a prognosis and is, therefore, important for the owners in terms of treatment expectations after radiation therapy [22]. As cribriform plate lysis is not always easy to detect, it is interesting that some c-CRP values were observed to be higher in dogs with T4 nasal tumors with cribriform plate lysis. Therefore, this marker could potentially be helpful in detecting or monitoring dogs of the T4 category. Due to the small number of dogs with the T4 category, further studies are needed to evaluate the c-CRP values in a larger group of these dogs.

The serum concentrations of the HPT of dogs in this study were within the reference interval of 0–3 g/L and were not significantly different among the different ND groups. This contradicts other findings, where HPT levels were significantly different between dogs with nasal discharge and healthy dogs as well as between dogs with aspergillosis and rhinitis [14]. The median values in the aforementioned study were higher than in the present study, with the median for aspergillosis at 5.2 g/L (IQR: 2–19.2), for rhinitis at 2.9 g/L (IQR: 0.8–5.8), for neoplasia at 4.1 g/L (IQR: 0.3–10.8), and in healthy dogs at 0.65 g/L (IQR: <0.02–4.3) [14]. The results of the present study may have been influenced by the measuring method. The cited study used an haptoglobin-hemoglobin-binding capacity assay [14,35] in contrast to the Tridelta PHASE Haptoglobin Assay [28] used in the present study. Other factors influencing the haptoglobin concentrations in the present study could have been the exclusion of dogs with additional diseases and the lower number of dogs with aspergillosis (3 dogs compared to the 13 dogs in the cited study) [14]. Furthermore, HPT could have been lower in the present study as dogs were excluded if pretreated with steroids 14 days before presentation in contrast to 7 days in the other study. Corticosteroids are reported to increase HPT concentrations [36], which has not been described for other APPs [10,36].

Circulating metabolite 25(OH)D is an indicator of vitamin D concentrations and plays an important role in the immune system [16]. The interpretation of serum concentrations may be difficult as the levels of 25(OH)D can vary widely in dogs [37]. Furthermore, its concentration is mainly determined by the amount of vitamin D intake through the diet, since dogs cannot adequately produce vitamin D3 in their skin under UV light [11,12]. Therefore, decreased 25(OH)D concentrations have been detected in dogs with various diseases, such as, e.g., chronic kidney disease, heart failure, inflammatory bowel disease, and mast cell tumors [11,12]. Interesting for the present study, recently, decreased concentrations have been detected in shelter dogs with infectious respiratory disease complex [38], as well as in dogs with sinonasal aspergillosis [17].

There was no significant difference in 25(OH)D concentrations between dogs with different NDs in this study. However, compared to the healthy control dogs, significantly lower 25(OH)D levels were detected in dogs with malignant tumors, especially sarcomas. This is in line with the literature, as 25(OH)D is discussed as a precursor of 1.25-dihydroxyvitamin D in tumorigenesis in humans [39,40], and, as in dogs with lower 25(OH)D concentrations, the relative risk of cancer was increased [37]. If vitamin D insufficiency is confirmed in further studies with larger numbers of dogs, vitamin D supplementation could possibly also prove useful in dogs with nasal tumors as supplementation is suggested to increase the responsiveness of cancer cells to chemotherapy [37] and to inhibit tumor cell growth [41]. Nevertheless, systemic vitamin D supplements for tumor therapy and prevention should be performed with caution, as it is associated with a risk of hypercalcemia [39,41]. 

Even if in the present study all 25(OH)D values were still within the reference interval of our external laboratory, all dogs showed concentrations beneath 250 nmol/L (=100 ng/mL). In the literature, 250 nmol/L was considered to be the minimum for vitamin D sufficiency (using a direct, competitive 25(OH)D chemiluminescence immunoassay and serum samples) via simultaneous measurements of intact parathyroid hormone [37] with deficient levels < 75 nmol/L (=30 ng/mL), insufficient concentrations with values between 75 and 250 nmol/L (=30–100 ng/mL), and sufficient levels above 250 nmol/L [37]. According to these data, all dogs in the present study, including controls, showed 25(OH)D insufficiency. Furthermore, 11 dogs with nasal disease (11/55; 20%) were deficient, with values < 75 nmol/L (two carcinomas, three sarcomas, two IR, and four others). One limitation is the low number of dogs with SNA, which is why 25(OH)D could not be examined separately in this group of dogs. 

However, if we would not have had to rule out the three dogs with primary SNA due to the above-mentioned limitations, and if we calculate the concentrations of the serum marker separately for all six dogs with SNA (three excluded primary SNA, two secondary SNA, and one primary SNA of the others group), the c-CRP (median 5.6 [IQR: 3.1–17.0]) and haptoglobin (median 1.4 [IQR 1.1–1.9]) concentrations were not statistically significantly different as were the 25(OH)D concentrations. However, in accordance with another study [17], the 25(OH)D concentrations appeared numerically low, with a median of 57.1 [IQR 51.9–150.7]. Therefore, further studies are needed to evaluate this finding in dogs with SNA.

The exact reasons for these low 25(OH)D concentrations in the present study, apart from the disease itself, cannot be given here. Another study also showed significant differences in 25(OH)D concentrations in apparently healthy dogs, with a few showing very low concentrations [37]. The control dogs in the present study were experimental dogs with a standardized diet, which may have had an influence here, but this cannot be determined retrospectively due to a lack of food analyses.

As with c-CRP and haptoglobin, differences in 25(OH)D concentrations may also be due to the measurement method. Although the measurement method used here was validated in the external laboratory for canine samples, there are no published studies in dogs that used this method and could be used for the comparison of concentrations.

In this study, we observed a negative correlation between c-CRP and 25(OH)D. This result is comparable to findings in humans, where it was shown that in addition to an increase in c-CRP, a reciprocal decrease in 25(OH)D may be observed [42,43,44]. Therefore, 25(OH)D was described as a negative acute-phase factor [42,43,44]. 

In the present study, nasal cavity diseases were diagnosed using standardized, comprehensive diagnostics. Dogs with other obvious diseases except the nasal cavity following whole-body CT with contrast and blood tests possibly influencing marker concentrations were excluded. It can be considered a limitation, as discussed before, that because the acute phase response is a rapidly occurring, non-specific reaction to an inflammatory stimulus, taking place even before the clinical signs of disease appear, significantly different initial changes in the included dogs may have been missed even if an increase in APPs has also been described under chronic inflammation [10,32]. As a further limitation, all dogs with nasal discharge were recruited at a tertiary institution, and selection bias cannot be excluded. To date, no significant influence of age, breed, or sex on the concentration of APPs has been found [10], which is why gender and age differences in the examined groups were not assumed to influence the results. 

## 5. Conclusions

The acute phase proteins c-CRP and HPT and the serum marker 25(OH)D were not significantly different among the groups of dogs with nasal cavity disease in the present study and are, therefore, not clinically useful for the reliable detection of nasal cavity disease and consequently not for therapy monitoring.

## Figures and Tables

**Table 1:** Concentrations of serum biomarkers canine C-reactive protein (c-CRP), haptoglobin (HPT), and 25-hydroxyvitamin-D (25(OH)D) in dogs with nasal cavity diseases (NDs) and controls. Indicated are the non-parametric data with median and interquartile range (IQR; in square brackets). Note: All median 25(OH)D values were within the reference interval regarding our external laboratory, but as the values are below 250 nmol/L (=100 ng/mL 25(OH)D), they are considered to be insufficient regarding other studies [11,12,29]. IR = idiopathic rhinitis.

		Dogs with Nasal Cavity Disease (n = 55)							Control Group (n = 10)	Excluded Dogs (n = 17)
	Reference Interval	Malignant NDs 25/55 Dogs	Carcinomas 13/55 Dogs	Sarcomas 12/55 Dogs	Benign NDs 30/55 Dogs	Benign Tumors 7/55 Dogs	IR 13/55 Dogs	Others 10/55 Dogs	Healthy 10 Dogs	With chronic/systemic diseases, and/or recent steroid therapy
c-CRP	up to 15 mg/L	4.3 [IQR: 1.4–8.8]	4.5 [IQR: 2.2–18.3]	3.3 [IQR: 0.6–8.9]	3.1 [IQR: 1.3–8]	2.5 [IQR: 1.1–4.6]	4.9 [IQR: 2.1–8.8]	3 [IQR: 0.4–8]	0.2 [IQR: 0–1.4]	4.3 [IQR: 1.8–36.4]
HPT	up to 3 g/L	1.5 [IQR: 1.1–1.9]	1.6 [IQR: 1.1–1.9]	1.5 [IQR: 1.1–1.7]	1.6 [IQR: 1.1–1.9]	1.3 [IQR: 0.8–1.5]	1.6 [IQR: 1.3–1.9]	1.6 [IQR: 1.3–2.0]	1.4 [IQR: 0.3–1.7]	1.5 [IQR: 1.3–1.8]
25(OH)D	48–350 nmol/L	112 [IQR: 90.3–149.5]	114.8 [IQR: 95.2–163.5]	103.6 [IQR: 77.2–144.5]	119 [IQR: 98.6–149.3]	134 [IQR: 99.7–146.6]	119.1 [IQR: 105–149.1]	112.6 [IQR: 57.6–171]	138.4 [IQR: 128.7–174.3]	108.3 [IQR: 53.5–169.4]

Columns containing data of combined groups of malignant and benign diseases are marked in a light gray color in the table header, as well as the column with data of excluded dogs due to various additional different systemic and/or chronic diseases or pre-treatment with steroids.

## Data Availability

The datasets used and/or analyzed during the current study are available from the corresponding author on reasonable request.

## Supplementary Materials

The following supporting information can be downloaded at: https://www.mdpi.com/article/10.3390/ani14192908/s1, Table S1: The results of the microbiological, histopathological examination as well as concentrations of detected serum markers in the included 55 dogs with nasal cavity disease (ND) are listed. The dogs are displayed in their groups according to the diagnosis of ND (idiopathic rhinitis, carcinoma, sarcoma, dogs of the others group, benign tumors). Orange marked fields in the table indicate dogs that have received antibiotics within weeks prior to presentation (n = 11). Green table fields indicate dogs (all with malignant nasal tumors) that have only had a culture-based microbiological examination as part of their medical history (n = 1) or that have not had one at all for financial restrictions (n = 3). Dark orange indicates an unknown antibiotic history (n = 1). In dogs with tumor diseases (malignant and benign) the T-category according to Adams et al. [22] is given.

## Abbreviations

AGR	Albumin-to-globulin ratio
APPs	Acute phase proteins
C	Carcinoma
c-CRP	Canine C-reactive protein
CG	Control group
CT	Computed tomography
ENT	Ear, nose, and throat
FB	Foreign bodies
HPT	Haptoglobin
IQR	Interquartile Range
IR	Idiopathic rhinitis
NLR	Neutrophil-to-lymphocyte ratio
ND	Nasal cavity disease
25(OH)D	25-hydroxyvitamin-D
O	Others
OND	Oronasal defects; connections between the oral and nasal cavities, such as cleft palate, periodontopathies, oronasal fistulas
PLR	Platelet-to-lymphocyte ratio
S	Sarcoma
SD	Standard deviation
SNA	Sinonasal aspergillosis
