# Supplementary material for: Diagnostic Utility of Canine C-Reactive Protein, Haptoglobin, and 25-Hydroxyvitamin-D in Dogs with Nasal Cavity Disease
Source: Animals (Basel). 2024 Oct 9;14(19):2908. doi: 10.3390/ani14192908 (PMC11475234; doi:10.3390/ani14192908)
Supplement: Supplementary file 1 [file animals-14-02908-s001.zip › animals-3158806-supplementary.pdf]

Supplementary Table S1. The results of the microbiological, histopathological examination as well as concentrations of detected serum markers in the included 55 dogs with nasal cavity disease (ND) are listed. The dogs are displayed in their groups according to the diagnosis of ND (idiopathic rhinitis, carcinoma, sarcoma, dogs of the others group, benign tumors). Orange marked fields in the table indicate dogs that have received antibiotics within 4 weeks prior to presentation (n=11). Green table fields indicate dogs (all with malignant nasal tumors) that have only had a culture-based microbiological examination as part of their medical history (n=1) or that have not had one at all for financial restrictions (n=3). Dark orange indicates an unknown antibiotic history (n=1). In dogs with tumor diseases (malignant and benign) the T-category according to Adams et al. [22] is given.

Consecutive number	Animal number in the experiment	Group/ Diagnosis of ND	Anti-biotic treatment	Antibiotic	Duration of antibiotic treatment	When was the antibiotic treatment given in relation to the presentation and diagnostics?	Result of culture based micro-biological examination of a nasal swab	Main inflammatory cell type in the histopathological examination of biopsies of nasal mucosa OR tumor type	T-category of nasal tumor	c-CRP concentration	25(OH)D concentration	Haptoglobin concentration
1	1	Idiopathic rhinitis	Yes	Amoxicillin Clavulanic Acid	4 weeks	6-8 weeks before presentation	Pasteurella multocida (high bacterial count)	Lympho-plasmacytic rhinitis	*Rhinitis-No grouping*	20.7	117.84	2.05
2	6	Idiopathic rhinitis	Yes	Doxycycline	3 weeks	Up to 2 weeks before presentation	Pasteurella canis (moderate bacterial count)	Chronic rhinitis	*Rhinitis-No grouping*	4.30	97.01	1.93
3	9	Idiopathic rhinitis	Yes	Amoxicillin Clavulanic Acid	3 weeks	Up to 8 weeks before presentation	Staphylococcus aureus (high bacterial count), Streptococcus canis (moderate	Chronic rhinitis	*Rhinitis-No grouping*	2.50	140.66	1.83

							bacterial count)					
4	10	Idiopathic rhinitis	Yes	Enrofloxacin	8 weeks	12 months before presentation	ß-hemolytic Strepto-coccus (moderate bacterial count, pure culture)	Neutrophilic rhinitis	*Rhinitis-No grouping*	4.90	112.96	1.60
5	18	Idiopathic rhinitis	Yes	Unknown	3 weeks	Up to 4 weeks before presentation	Negative	Chronic rhinitis	*Rhinitis-No grouping*	2.1	134.81	1.6
6	23	Idiopathic rhinitis	Yes	Amoxicillin Clavulanic Acid	1 week	Up to 4 weeks before presentation	Staphylo-coccus aureus (moderate bacterial count, pure culture)	Lympho-plasmacytic rhinitis	*Rhinitis-No grouping*	21.3	160.2	1.76
7	24	Idiopathic rhinitis	Yes	Doxycycline	1 week	Up to 5 days before presentation	Negative	Chronic rhinitis	*Rhinitis-No grouping*	0	119.11	1.41
8	26	Idiopathic rhinitis	Yes	Amoxicillin Clavulanic Acid	2 weeks	Up to 3 weeks before presentation	Staphylo-coccus intermedius (high bacterial count, pure culture)	Lympho-plasmacytic rhinitis	*Rhinitis-No grouping*	2	118.85	2.22
9	29	Idiopathic rhinitis	Yes	Doxycycline (two times), Amoxicillin Clavulanic Acid	unknown	4 years and 3 years before presentation	ß-hemolytic Strepto-coccus (high bacterial count)	Lympho-plasmacytic rhinitis	*Rhinitis-No grouping*	5.4	132.53	0.24
10	42	Idiopathic rhinitis	Yes	Doxycycline	3 weeks	5 months before presentation	Pasteurella multocida (moderate bacterial	Chronic rhinitis	*Rhinitis-No grouping*	0.1	157.54	1.1

							count, pure culture)					
11	45	Idiopathic rhinitis	Yes	Cefalexin (a), Doxycycline (b)	Unknown duration (a) and 8 days (b)	9 months before presentation (a) and 3 months before presentation (b)	Negative	Neutrophilic rhinitis	*Rhinitis-No grouping*	9.5	197.75	1.53
12	47	Idiopathic rhinitis	Yes	Doxycycline	3 weeks	11 months before presentation	Negative	Neutrophilic rhinitis	*Rhinitis-No grouping*	8.1	62.6	0.92
13	53	Idiopathic rhinitis	Yes	Amoxicillin Clavulanic Acid	Unknown	More than 4 weeks before presentation	Negative	Neutrophilic rhinitis	*Rhinitis-No grouping*	7.9	70.63	1.59
1	2	Carcinoma	No	No	No	No	Not performed	Carcinoma	4	5.70	47.97	1.82
2	4	Carcinoma	No	No	No	No	Strepto-coccus canis (high bacterial count), Staphylo-coccus intermedius (in high bacterial count)	Transitional cell carcinoma	2	5.60	101.34	1.71
3	7	Carcinoma	No	No	No	No	Negative	Adenocarcinoma	4	0.50	149.27	1.96
4	8	Carcinoma	Yes	Clindamycin and Amoxicillin Trihydrate	4 days each	Until some days before presentation	Negative	Transitional cell carcinoma	4	39.00	111.99	1.97
5	14	Carcinoma	No	No	No	No	Staphylo-coccus intermedius	Adenocarcinoma	4	29.30	102.62	1.56

							(high bacterial count, pure culture)					
6	26	Carcinoma	Yes	Marbofloxacin	Unknown	More than 4 weeks before presentation	ß-hemolytic Strepto-coccus (high bacterial count), Staphylo-coccus intermedius (high bacterial count)	Adenocarcinoma	4	4.1	176.92	1.61
7	33	Carcinoma	No	No	No	No	Strepto-coccus canis (high bacterial count)	Carcinoma	4	139.6	89.01	1.93
8	36	Carcinoma	No	No	No	No	Negative	Adenocarcinoma	1	1.8	136.03	0.63
9	40	Carcinoma	Unknown	Different medications but unknown if antibiotics (not to reevaluate)	Unknown	Unknown	Negative	Carcinoma in situ	1	0.9	169.11	0.92
10	42	Carcinoma	Yes	Marbofloxacin	3 weeks	Up to 4 weeks before presentation	Strepto-coccus canis (high bacterial count), Staphylo-coccus intermedius (high bacterial count)	Squamous cell carcinoma	3	4.5	114.81	1.25

11	47	Carcinoma	Yes	Two different unknown antibiotics	Unknown	More than 4 weeks before presentation	Staphylo-coccus intermedius (moderate bacterial count)	Carcinoma	2	4.5	59.3	1.11
12	49	Carcinoma	Yes	Amoxicillin Clavulanic Acid	5 days	2 months before presentation	Pre-reported to be negative (at the regular veterinarian)	Transitional cell carcinoma	1	2.5	157.82	1.15
13	51	Carcinoma	Yes	Amoxicillin Clavulanic Acid	3 days	Till presentation	Not performed	Carcinoma	4	7.3	185.68	1.89
1	3	Sarcoma	No	No	No	No	Negative	Chondrosarcoma	2	3.70	149.64	0.70
2	5	Sarcoma	Yes	Unknown	Unknown	More than 4 weeks before presentation	Staphylo-coccus intermedius (high bacterial count, pure culture)	Chondrosarcoma	3	3.00	120.12	1.54
3	12	Sarcoma	No	No	No	No	Not performed	Hemangio-sarcoma	1	2.60	60.18	1.51
4	15	Sarcoma	No	No	No	No	Staphylo-coccus intermedius (high bacterial count, pure culture)	Sarcoma	4	0.50	100.94	1.59
5	19	Sarcoma	No	No	No	No	Negative	Sarcoma	3	10.3	156.83	1.18
6	30	Sarcoma	No	No	No	No	Pasteurella canis (moderate	Chondrosarcoma	3	4.6	143.24	2.27

							bacterial count)					
7	31	Sarcoma	Yes	Marbofloxacin	2 weeks	Till presentation	Staphylo-coccus intermedius (moderate bacterial count, pure culture)	Chondrosarcoma	3	10.3	106.18	2.26
8	34	Sarcoma	Yes	Enrofloxacin	Unknown	More than 4 weeks before presentation	Negative	Osteosarcoma	3	0	144.94	1.49
9	35	Sarcoma	Yes	Multiple antibiotics	Unknown	More than 4 weeks before presentation	Negative	Sarcoma	3	1	72.44	1.42
10	38	Sarcoma	Yes	Unknown	Unknown	More than 4 weeks before presentation	Negative	Chondrosarcoma	4	4.3	30.34	1.13
11	47	Sarcoma	Yes	Unknown	Unknown	More than 4 weeks before presentation	Negative	Chondrosarcoma	4	21	91.57	1.04
12	49	Sarcoma	Yes	2 different unknown antibiotics	1 week each	Up to 2 weeks before presentation	Staphylo-coccus aureus (high bacterial count)	Chondrosarcoma	1	0.4	94.6	1.68
1	11	OTHERS: Traumatic rhinitis, parodonto-pathies, oronasal fistula, nasal outlet stenosis	Yes	Unknown	Unknown	More than 4 weeks before presentation	Pseudo-monas aeruginosa (low bacterial count, pure culture)	Chronic rhinitis	*Rhinitis-No grouping*	2.50	137.92	2.13

2	13	OTHERS: Primary SNA	Yes	Unknown	Unknown	More than 4 weeks before presentation	Negative; mycological examination: Aspergillus fumigatus	Necrotizing rhinitis	*Rhinitis-No grouping*	42.30	50.22	1.83
3	16	OTHERS: Nasal foreign body, parodonto-pathies	Yes	Doxycycline	3 weeks	Up to 8 weeks before presentation	Negative	Chronic rhinitis	*Rhinitis-No grouping*	3.1	60.11	2.28
4	17	OTHERS: Secondary SNA, nasal foreign body	Yes	Marbofloxaci n	Unknown	Up to one day before presentation	Negative; mycological examination: Aspergillus fumigatus	Plasmacytic rhinitis	*Rhinitis-No grouping*	2.9	225.57	1.91
5	21	OTHERS: Naso-pharyngeal foreign body	Yes	Unknown	2 weeks	Up to 2 weeks before presentation	Negative	Neutrophilic rhinitis	*Rhinitis-No grouping*	17.6	117.99	1.4
6	25	OTHERS: Nasal outlet stenosis, parodonto-pathies	Yes	Unknown	Unknown	More than 4 weeks before presentation	Negative	Lympho-plasmacytic rhinitis	*Rhinitis-No grouping*	0.5	184.7	1.57
7	28	OTHERS: Oronasal fistula	Yes	Amoxicillin Trihydrate and Marbofloxaci n	Both together for one week	Up to one week before presentation	Negative	Neutrophilic rhinitis	*Rhinitis-No grouping*	0	107.26	1.61
8	38	OTHERS: Rhinitis after confirmed and healed asper-gillosis	Yes	Amoxicillin Clavulanic Acid	10 days	Up to three weeks before presentation	Negative	Chronic rhinitis	*Rhinitis-No grouping*	0	166.41	0.99

9	40	OTHERS: Secondary asper-gillosis with nasal foreign body	Yes	Two unknown antibiotics	Unknown	More than 4 weeks before presentation	Klebsiella pneumoniae (moderate bacterial count)	Mycotic rhinitis	*Rhinitis-No grouping*	3.1	60.64	1.07
10	44	OTHERS: Oronasal fistula	Yes	Unknown	Unknown	More than 4 weeks before presentation	Staphylo-coccus intermedius (moderate bacterial count)	Chronic rhinitis	*Rhinitis-No grouping*	4.8	42.4	1.63
1	22	Benign tumor	Yes	Amoxicillin (2 times)	unknown	Up to 3 months before presentation	Negative	Polypous rhinitis	3	2.5	112.32	0.88
2	30	Benign tumor	Yes	Unknown antibiotic (a), Amoxicillin (b), Doxycycline (c)	Unknown (a), 7 days (b), 21 days (c)	Up to 9 months before presentation	Staphylo-coccus intermedius (moderate bacterial count)	Hamartoma	3	0	146.61	0.79
3	33	Benign tumor	No	No	No	No	Negative	Benign hyperplasia, glandular proliferation	1	1.4	99.73	1.31
4	46	Benign tumor	No	No	No	No	Negative	Benign proliferation	3	1.1	133.95	0.14
5	54	Benign tumor	Yes	Unknown	Unknown	More than 4 weeks before presentation	Negative	Polypous rhinitis	3	8.8	99.07	1.46
6	55	Benign tumor	No	No	No	No	Negative	Benign tumor	3	4.6	139.12	1.27

7	56	Benign tumor	Yes	Amoxicillin Clavulanic Acid	unknown	More than 4 weeks before presentation	Negative	Hamartoma	3	4.1	169.35	2.11
